# Crystal structure of (2-benzyl­oxy­pyrimidin-5-yl)boronic acid

**DOI:** 10.1107/S1600536814024519

**Published:** 2014-11-15

**Authors:** Krzysztof Durka, Tomasz Kliś, Janusz Serwatowski

**Affiliations:** a Physical Chemistry Department, Faculty of Chemistry, Warsaw University of Technology, Noakowskiego 3, 00-664 Warsaw, Poland

**Keywords:** crystal structure, aryl­boronic acid, hydrogen-bonding inter­actions

## Abstract

The boronic acid group in the title compound, C_11_H_11_BN_2_O_3_, adopts a *syn–anti* conformation and is almost coplanar with the aromatic rings , making a dihedralangle of 3.8 (2)°. In the crystal, adjacent mol­ecules are linked *via* pairs of O—H⋯O inter­actions, forming centrosymmetric dimers with an *R*_2_^2^(8) motif, which have recently been shown to be energetically very favorable (Durka *et al.*, 2012 ▶, 2014 ▶). The hy­droxy groups in an *anti* conformation are engaged in lateral hydrogen-bonding inter­actions with N atoms from neighbouring mol­ecules, leading to the formation of chains along [001]. O⋯B [3.136 (2) Å] and C(π)⋯B [3.393 (2) Å] stacking inter­actions in turn link parallel chains of centrosymmetric dimers into layers parallel to (010).

## Related literature   

For general background to the structures of boronic acids, see, for example: Hall (2011 ▶); Luliński *et al.* (2007 ▶); Maly *et al.* (2006 ▶); Shimpi *et al.* (2007 ▶). For the characterization of related pyrymidylboronic acids, see: Clapham *et al.* (2007 ▶); Liao *et al.* (1964 ▶); Peters *et al.* (1990 ▶); Saygili *et al.* (2004 ▶).
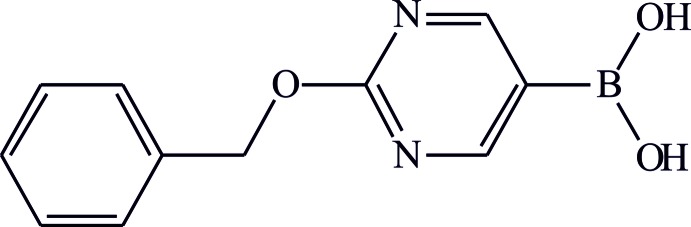


## Experimental   

### Crystal data  

C_11_H_11_BN_2_O_3_
*M* *_r_* = 230.03
Monoclinic, 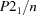

*a* = 5.498 (1) Å
*b* = 30.4320 (17) Å
*c* = 6.7086 (19) Å
β = 113.54 (4)°
*V* = 1029.0 (4) Å^3^
*Z* = 4
Mo *K*α radiation
μ = 0.11 mm^−1^
*T* = 100 K
0.20 × 0.15 × 0.15 mm

### Data collection  

Kuma KM-4 CCD diffractometer
Absorption correction: multi-scan (*CrysAlis PRO*; Agilent, 2013 ▶) *T*_min_ = 0.993, *T*_max_ = 1.000
18472 measured reflections
4167 independent reflections
2975 reflections with *I* > 2σ(*I*)
*R*_int_ = 0.044

### Refinement  

*R*[*F* ^2^ > 2σ(*F* ^2^)] = 0.053
*wR*(*F* ^2^) = 0.135
*S* = 1.03
4167 reflections
160 parameters
H atoms treated by a mixture of independent and constrained refinement
Δρ_max_ = 0.57 e Å^−3^
Δρ_min_ = −0.30 e Å^−3^

### 

Data collection: *CrysAlis PRO* (Agilent, 2013 ▶); cell refinement: *CrysAlis PRO*; data reduction: *CrysAlis PRO*; program(s) used to solve structure: *SHELXS97* (Sheldrick, 2008 ▶); program(s) used to refine structure: *SHELXL2013* (Sheldrick, 2008 ▶); molecular graphics: *DIAMOND* (Brandenburg, 2005 ▶); software used to prepare material for publication: *publCIF* (Westrip, 2010 ▶).

## Supplementary Material

Crystal structure: contains datablock(s) I. DOI: 10.1107/S1600536814024519/zl2604sup1.cif

Structure factors: contains datablock(s) I. DOI: 10.1107/S1600536814024519/zl2604Isup2.hkl

Supporting information file. DOI: 10.1107/S1600536814024519/zl2604Isup3.mol

Supporting information file. DOI: 10.1107/S1600536814024519/zl2604Isup4.cml

**Fig. 1.**
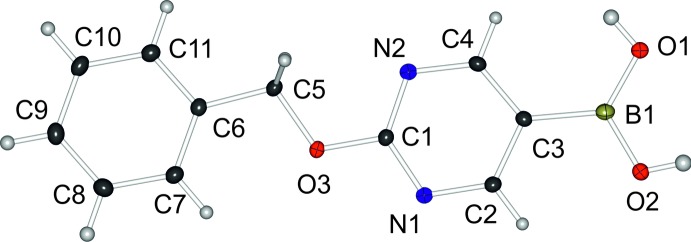
Labelling of atoms and estimation of their atomic thermal motion as Anisotropic Displacement Parameters (ADPs, 50% probability level) for 1.

1 . DOI: 10.1107/S1600536814024519/zl2604fig1.tif
Labelling of atoms and estimation of their atomic thermal motion as Anisotropic Displacement Parameters (ADPs, 50% probability level) for **1**.

**Fig. 2.**
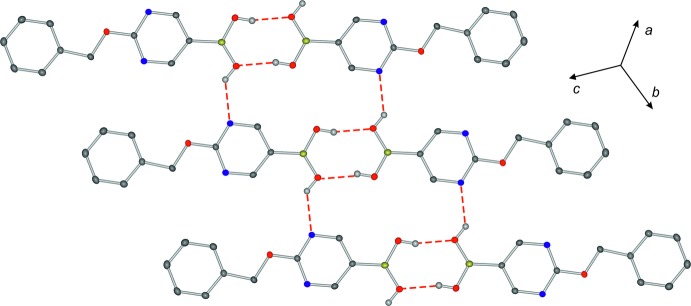
The molecular chains in 1. Hydrogen bonds are shown as red, dashed lines. Aromatic and aliphatic hydrogen atoms are omitted for clarity.

1 . DOI: 10.1107/S1600536814024519/zl2604fig2.tif
The mol­ecular chains in **1**. Hydrogen bonds are shown as red, dashed lines. Aromatic and aliphatic hydrogen atoms are omitted for clarity.

**Fig. 3.**
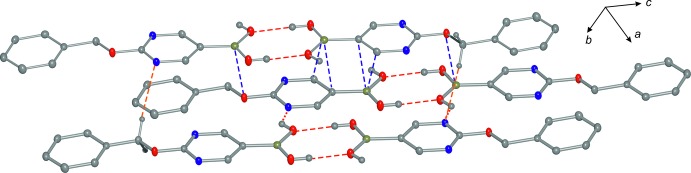
Structural graph displaying the intermolecular O···B, C(π)···B (blue), C—H···N (orange) and O—H···O, O—H···N (red) interactions.

. DOI: 10.1107/S1600536814024519/zl2604fig3.tif
Structural graph displaying the inter­molecular O⋯B, C(π)⋯B (blue), C—H⋯N (orange) and O—H⋯O, O—H⋯N (red) inter­actions.

CCDC reference: 937427

Additional supporting information:  crystallographic information; 3D view; checkCIF report

## Figures and Tables

**Table 1:** Hydrogen-bond geometry (, )

*D*H*A*	*D*H	H*A*	*D* *A*	*D*H*A*
C2H2O2^i^	0.95	2.60	3.5104(15)	161
O2H2*A*O1^ii^	0.852(19)	1.915(19)	2.7615(16)	172.7(17)
O1H1*A*N1^iii^	0.849(18)	2.067(18)	2.8188(15)	147.2(16)

Symmetry codes: (i) 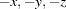; (ii) 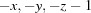; (iii) 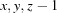.

## supplementary crystallographic information

### S1. Comment

Heterocyclic boronic acids have been intensively studied in the recent years. They have found numerous application as Suzuki-Miyaura cross-coupling partners. However, pyrimidylboronic acids have been largely neglected, although some derivatives were synthesized (Clapham *et al.*, 2007; Liao *et al.*, 1964; Peters *et al.*, 1990; Saygili *et al.*, 2004). In this manuscript we focus our attention on a pyrimidine boronic acid derivative containing a benzyl­oxy group at the 2^nd^ position.

The molecular structure of 1 shows that the B(OH)_2_ group adopts the usual *syn-anti* conformation. The entire molecule including both aromatic rings and boronic group remains essentially planar (Figure 1). On the first level of material organization, centrosymmetric O—H···O hydrogen-bonded dimers are formed. They are linked *via* lateral hydrogen-bonding inter­actions with nitro­gen atoms from neighbouring molecules. It results in the formation of molecular chains propagated along the [001] direction (Figure 2). Molecules from adjacent chains inter­act by weak O···B and C(π)···B stacking inter­actions, which link parallel oriented chains into 2D layers. These contacts are additionally supported by weak C—H···N inter­actions between the methyl­ene group and one of the N atoms of the pyrimidyl ring, and also by C—H···O inter­actions formed between the C(pyrimidyl)—H group and an oxygen atom from the B(OH)_2_ group. The supra­molecular architecture extends further due to weak C—H···C(π) contacts leading to a three-dimensional network.

### S2. Crystallization

The title compound was received from Aldrich. Crystals suitable for single-crystal X-ray diffraction analysis were grown by cooling a solution of the boronic acid (0.2 g) in acetone (4 ml).

### S3. Refinement

Crystal data, data collection and structure refinement details are summarized in Table 1. All CH (methyl­ene, phenyl) hydrogen atoms were placed in calculated positions with C—H distances of 0.95Å (phenyl) and 0.99Å (methyl­ene). They were included in the refinement in riding-motion approximation with U_iso_(phenyl H)=1.2U_eq_(C), and U_iso_(methyl H) = 1.5U_eq_(C). The positions of OH hydrogen atoms were refined with U_iso_(hy­droxy H) = 1.5U_eq_(C).

### Crystal data

**Table:**

C_11_H_11_BN_2_O_3_	*F*(000) = 480
*M**_r_* = 230.03	*D*_x_ = 1.485 Mg m^−^^3^
Monoclinic, *P*2_1_/*n*	Mo *K*α radiation, λ = 0.71073 Å
*a* = 5.498 (1) Å	Cell parameters from 4979 reflections
*b* = 30.4320 (17) Å	θ = 2.0–34.4°
*c* = 6.7086 (19) Å	µ = 0.11 mm^−^^1^
β = 113.54 (4)°	*T* = 100 K
*V* = 1029.0 (4) Å^3^	Unspecified, colourless
*Z* = 4	0.20 × 0.15 × 0.15 mm

### Data collection

**Table:**

Kuma KM-4 CCD diffractometer	4167 independent reflections
Radiation source: fine-focus sealed tube	2975 reflections with *I* > 2σ(*I*)
Graphite monochromator	*R*_int_ = 0.044
ω scan	θ_max_ = 34.5°, θ_min_ = 2.7°
Absorption correction: multi-scan (*CrysAlis PRO*; Agilent, 2013)	*h* = −8→8
*T*_min_ = 0.993, *T*_max_ = 1.000	*k* = −48→47
18472 measured reflections	*l* = −10→10

### Refinement

**Table:**

Refinement on *F*^2^	0 restraints
Least-squares matrix: full	Hydrogen site location: mixed
*R*[*F*^2^ > 2σ(*F*^2^)] = 0.053	H atoms treated by a mixture of independent and constrained refinement
*wR*(*F*^2^) = 0.135	*w* = 1/[σ^2^(*F*_o_^2^) + (0.0561*P*)^2^ + 0.4141*P*] where *P* = (*F*_o_^2^ + 2*F*_c_^2^)/3
*S* = 1.03	(Δ/σ)_max_ < 0.001
4167 reflections	Δρ_max_ = 0.57 e Å^−^^3^
160 parameters	Δρ_min_ = −0.30 e Å^−^^3^

### Special details

**Table:**

Geometry. All e.s.d.'s (except the e.s.d. in the dihedral angle between two l.s. planes) are estimated using the full covariance matrix. The cell e.s.d.'s are taken into account individually in the estimation of e.s.d.'s in distances, angles and torsion angles; correlations between e.s.d.'s in cell parameters are only used when they are defined by crystal symmetry. An approximate (isotropic) treatment of cell e.s.d.'s is used for estimating e.s.d.'s involving l.s. planes.
Refinement. Refinement of *F*^2^ against ALL reflections. The weighted *R*-factor *wR* and goodness of fit *S* are based on *F*^2^, conventional *R*-factors *R* are based on *F*, with *F* set to zero for negative *F*^2^. The threshold expression of *F*^2^ > σ(*F*^2^) is used only for calculating *R*-factors(gt) *etc*. and is not relevant to the choice of reflections for refinement. *R*-factors based on *F*^2^ are statistically about twice as large as those based on *F*, and *R*- factors based on all data will be even larger.

### Fractional atomic coordinates and isotropic or equivalent isotropic displacement parameters (Å^2^)

**Table:**

	*x*	*y*	*z*	*U*_iso_*/*U*_eq_	
C1	0.7668 (2)	0.09979 (4)	0.33931 (19)	0.0119 (2)	
C2	0.4044 (2)	0.05846 (4)	0.16000 (19)	0.0129 (2)	
H2	0.2541	0.0426	0.1570	0.016*	
C3	0.4464 (2)	0.06065 (4)	−0.03164 (19)	0.0120 (2)	
C4	0.6720 (2)	0.08390 (4)	−0.01318 (19)	0.0135 (2)	
H4	0.7143	0.0856	−0.1371	0.016*	
C5	1.1273 (2)	0.14700 (4)	0.5176 (2)	0.0144 (2)	
H5A	1.0515	0.1694	0.4023	0.017*	
H5B	1.2516	0.1286	0.4803	0.017*	
C6	1.2746 (2)	0.16928 (4)	0.73226 (19)	0.0119 (2)	
C7	1.1923 (2)	0.16861 (4)	0.9030 (2)	0.0152 (2)	
H7	1.0405	0.1521	0.8908	0.018*	
C8	1.3326 (3)	0.19224 (4)	1.0918 (2)	0.0178 (2)	
H8	1.2757	0.1916	1.2081	0.021*	
C9	1.5544 (3)	0.21671 (4)	1.1128 (2)	0.0170 (2)	
H9	1.6466	0.2333	1.2410	0.020*	
C10	1.6401 (3)	0.21665 (4)	0.9434 (2)	0.0161 (2)	
H10	1.7933	0.2329	0.9567	0.019*	
C11	1.5022 (2)	0.19294 (4)	0.7556 (2)	0.0143 (2)	
H11	1.5630	0.1928	0.6415	0.017*	
B1	0.2490 (3)	0.03690 (4)	−0.2435 (2)	0.0129 (2)	
N1	0.5624 (2)	0.07726 (3)	0.34800 (16)	0.0133 (2)	
N2	0.8334 (2)	0.10406 (4)	0.16971 (17)	0.0137 (2)	
O1	0.28595 (18)	0.03484 (3)	−0.43207 (14)	0.01608 (19)	
O2	0.03654 (19)	0.01745 (3)	−0.22897 (15)	0.0177 (2)	
O3	0.91719 (17)	0.11992 (3)	0.52723 (14)	0.01468 (18)	
H1A	0.416 (3)	0.0477 (6)	−0.445 (3)	0.022*	
H2A	−0.068 (3)	0.0034 (6)	−0.340 (3)	0.022*	

### Atomic displacement parameters (Å^2^)

**Table:**

	*U*^11^	*U*^22^	*U*^33^	*U*^12^	*U*^13^	*U*^23^
C1	0.0114 (5)	0.0120 (5)	0.0124 (5)	−0.0016 (4)	0.0048 (4)	−0.0011 (4)
C2	0.0135 (5)	0.0138 (5)	0.0122 (5)	−0.0028 (4)	0.0059 (4)	−0.0002 (4)
C3	0.0129 (5)	0.0125 (5)	0.0112 (5)	−0.0013 (4)	0.0054 (4)	−0.0001 (4)
C4	0.0150 (5)	0.0157 (5)	0.0119 (5)	−0.0016 (4)	0.0074 (4)	−0.0008 (4)
C5	0.0134 (5)	0.0163 (5)	0.0144 (5)	−0.0052 (4)	0.0066 (4)	−0.0024 (4)
C6	0.0112 (5)	0.0111 (5)	0.0128 (5)	−0.0006 (4)	0.0039 (4)	−0.0003 (4)
C7	0.0145 (5)	0.0163 (5)	0.0159 (5)	−0.0030 (4)	0.0073 (5)	−0.0007 (4)
C8	0.0201 (6)	0.0202 (6)	0.0149 (5)	−0.0038 (5)	0.0088 (5)	−0.0018 (5)
C9	0.0167 (6)	0.0169 (6)	0.0152 (5)	−0.0021 (4)	0.0040 (5)	−0.0029 (4)
C10	0.0121 (5)	0.0165 (6)	0.0183 (6)	−0.0027 (4)	0.0046 (5)	−0.0012 (4)
C11	0.0136 (5)	0.0149 (5)	0.0153 (5)	−0.0008 (4)	0.0067 (4)	0.0002 (4)
B1	0.0134 (6)	0.0125 (6)	0.0133 (6)	0.0003 (4)	0.0060 (5)	0.0019 (5)
N1	0.0130 (5)	0.0154 (5)	0.0119 (4)	−0.0033 (4)	0.0054 (4)	−0.0013 (4)
N2	0.0141 (5)	0.0157 (5)	0.0125 (4)	−0.0035 (4)	0.0064 (4)	−0.0016 (4)
O1	0.0171 (4)	0.0204 (4)	0.0126 (4)	−0.0069 (3)	0.0079 (3)	−0.0029 (3)
O2	0.0179 (4)	0.0240 (5)	0.0124 (4)	−0.0093 (4)	0.0071 (3)	−0.0041 (4)
O3	0.0148 (4)	0.0175 (4)	0.0121 (4)	−0.0069 (3)	0.0057 (3)	−0.0036 (3)

### Geometric parameters (Å, º)

**Table:**

C1—N2	1.3336 (15)	C6—C11	1.3972 (16)
C1—N1	1.3375 (15)	C7—C8	1.3912 (18)
C1—O3	1.3474 (15)	C7—H7	0.9500
C2—N1	1.3407 (16)	C8—C9	1.3870 (18)
C2—C3	1.3957 (17)	C8—H8	0.9500
C2—H2	0.9500	C9—C10	1.3933 (19)
C3—C4	1.3898 (16)	C9—H9	0.9500
C3—B1	1.5775 (19)	C10—C11	1.3856 (18)
C4—N2	1.3415 (16)	C10—H10	0.9500
C4—H4	0.9500	C11—H11	0.9500
C5—O3	1.4412 (14)	B1—O2	1.3475 (16)
C5—C6	1.5028 (18)	B1—O1	1.3602 (16)
C5—H5A	0.9900	O1—H1A	0.849 (18)
C5—H5B	0.9900	O2—H2A	0.852 (19)
C6—C7	1.3898 (17)		
			
N2—C1—N1	127.51 (11)	C6—C7—H7	120.1
N2—C1—O3	118.63 (10)	C8—C7—H7	120.1
N1—C1—O3	113.86 (10)	C9—C8—C7	121.03 (12)
N1—C2—C3	124.24 (11)	C9—C8—H8	119.5
N1—C2—H2	117.9	C7—C8—H8	119.5
C3—C2—H2	117.9	C8—C9—C10	119.09 (12)
C4—C3—C2	114.36 (11)	C8—C9—H9	120.5
C4—C3—B1	125.58 (11)	C10—C9—H9	120.5
C2—C3—B1	120.05 (10)	C11—C10—C9	120.12 (12)
N2—C4—C3	123.72 (11)	C11—C10—H10	119.9
N2—C4—H4	118.1	C9—C10—H10	119.9
C3—C4—H4	118.1	C10—C11—C6	120.71 (12)
O3—C5—C6	110.46 (10)	C10—C11—H11	119.6
O3—C5—H5A	109.6	C6—C11—H11	119.6
C6—C5—H5A	109.6	O2—B1—O1	120.19 (12)
O3—C5—H5B	109.6	O2—B1—C3	116.12 (11)
C6—C5—H5B	109.6	O1—B1—C3	123.67 (11)
H5A—C5—H5B	108.1	C1—N1—C2	114.70 (10)
C7—C6—C11	119.14 (11)	C1—N2—C4	115.40 (10)
C7—C6—C5	123.72 (11)	B1—O1—H1A	121.3 (12)
C11—C6—C5	117.11 (11)	B1—O2—H2A	117.4 (12)
C6—C7—C8	119.86 (11)	C1—O3—C5	114.90 (9)
			
N1—C2—C3—C4	0.78 (18)	C4—C3—B1—O2	−177.72 (12)
N1—C2—C3—B1	179.43 (11)	C2—C3—B1—O2	3.78 (17)
C2—C3—C4—N2	−2.18 (18)	C4—C3—B1—O1	3.3 (2)
B1—C3—C4—N2	179.25 (12)	C2—C3—B1—O1	−175.21 (12)
O3—C5—C6—C7	−8.77 (17)	N2—C1—N1—C2	−2.59 (19)
O3—C5—C6—C11	173.27 (10)	O3—C1—N1—C2	177.44 (10)
C11—C6—C7—C8	1.58 (19)	C3—C2—N1—C1	1.38 (18)
C5—C6—C7—C8	−176.34 (12)	N1—C1—N2—C4	1.33 (19)
C6—C7—C8—C9	0.2 (2)	O3—C1—N2—C4	−178.69 (11)
C7—C8—C9—C10	−1.5 (2)	C3—C4—N2—C1	1.25 (18)
C8—C9—C10—C11	0.98 (19)	N2—C1—O3—C5	4.30 (16)
C9—C10—C11—C6	0.78 (19)	N1—C1—O3—C5	−175.72 (10)
C7—C6—C11—C10	−2.06 (18)	C6—C5—O3—C1	177.64 (10)
C5—C6—C11—C10	176.00 (11)		

### Hydrogen-bond geometry (Å, º)

**Table:**

*D*—H···*A*	*D*—H	H···*A*	*D*···*A*	*D*—H···*A*
C2—H2···O2^i^	0.95	2.60	3.5104 (15)	161
O2—H2*A*···O1^ii^	0.852 (19)	1.915 (19)	2.7615 (16)	172.7 (17)
O1—H1*A*···N1^iii^	0.849 (18)	2.067 (18)	2.8188 (15)	147.2 (16)

Symmetry codes: (i) −*x*, −*y*, −*z*; (ii) −*x*, −*y*, −*z*−1; (iii) *x*, *y*, *z*−1.

## References

[bb1] Agilent (2013). *CrysAlis PRO*. Agilent Technologies, Santa Clara, USA.

[bb2] Brandenburg, K. (2005). *DIAMOND*. Crystal Impact GbR, Bonn, Germany.

[bb3] Clapham, K. M., Smith, A. E., Batsanov, A. S., McIntyre, L., Pountney, A., Bryce, M. R. & Tarbit, B. (2007). *Eur. J. Org. Chem.* pp. 5712–5716.

[bb4] Durka, K., Jarzembska, K. N., Kamiński, R., Luliński, S., Serwatowski, J. & Woźniak, K. (2012). *Cryst. Growth Des.* **12**, 3720–3734.

[bb5] Durka, K., Luliński, S., Jarzembska, K. N., Smętek, J., Serwatowski, J. & Woźniak, K. (2014). *Acta Cryst.* B**70**, 157–171. 10.1107/S2052520613034987 24441139

[bb6] Hall, D. G. (2011). *Boronic Acids*, pp. 3–8. Weinheim: Wiley-VCH.

[bb7] Liao, T. K., Podrebarac, E. G. & Cheng, C. C. (1964). *J. Am. Chem. Soc.* **86**, 1869–1870.

[bb8] Luliński, S., Madura, I., Serwatowski, J., Szatyłowicz, H. & Zachara, J. (2007). *New J. Chem.* **31**, 144–15.

[bb9] Maly, K. E., Maris, T. & Wuest, J. D. (2006). *CrystEngComm*, **8**, 33–35.

[bb10] Peters, D., Hörnfeldt, A. B. & Gronowitz, S. (1990). *J. Heterocycl. Chem.* **27**, 2165–2173.

[bb11] Saygili, N., Batsanov, A. S. & Bryce, M. R. (2004). *Org. Biomol. Chem.* **2**, 852–857. 10.1039/b314624n 15007413

[bb12] Sheldrick, G. M. (2008). *Acta Cryst.* A**64**, 112–122. 10.1107/S0108767307043930 18156677

[bb13] Shimpi, M. R., SeethaLekshmi, N. & Pedireddi, V. R. (2007). *Cryst. Growth Des.* **7**, 1958–1963.

[bb14] Westrip, S. P. (2010). *J. Appl. Cryst.* **43**, 920–925.

