# Supplementary figures and images for: Crystal structure of (2-benzyl­oxy­pyrimidin-5-yl)boronic acid
Source: Acta Crystallogr Sect E Struct Rep Online. 2014 Nov 15;70(Pt 12):o1259–60. doi: 10.1107/S1600536814024519 (PMC4257459; doi:10.1107/S1600536814024519)

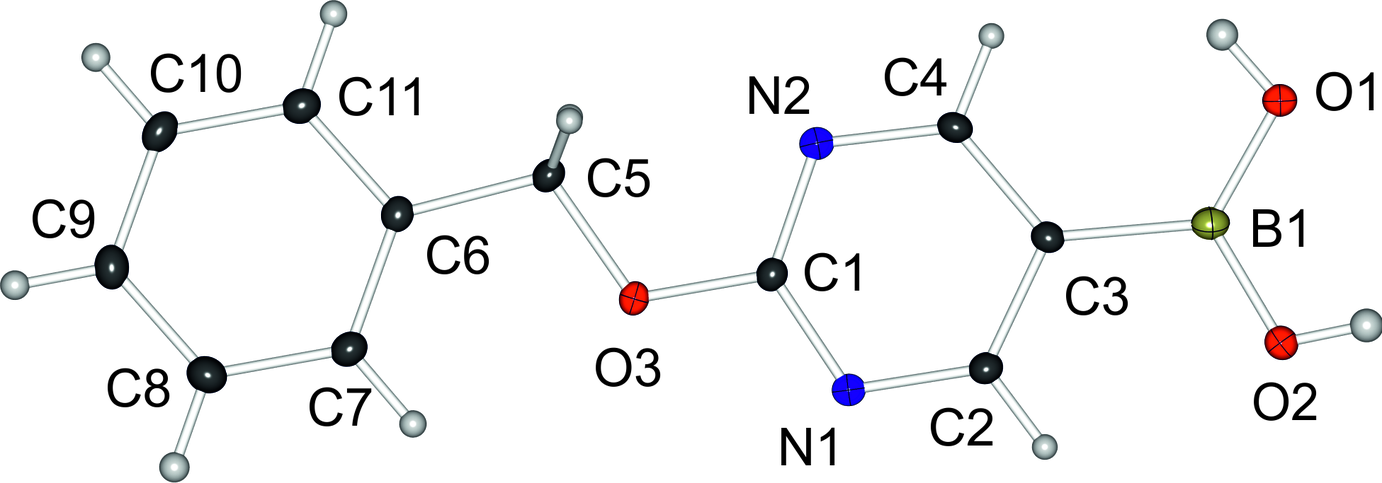

Supplement: Supplementary file 5 [file e-70-o1259-fig1.tif]

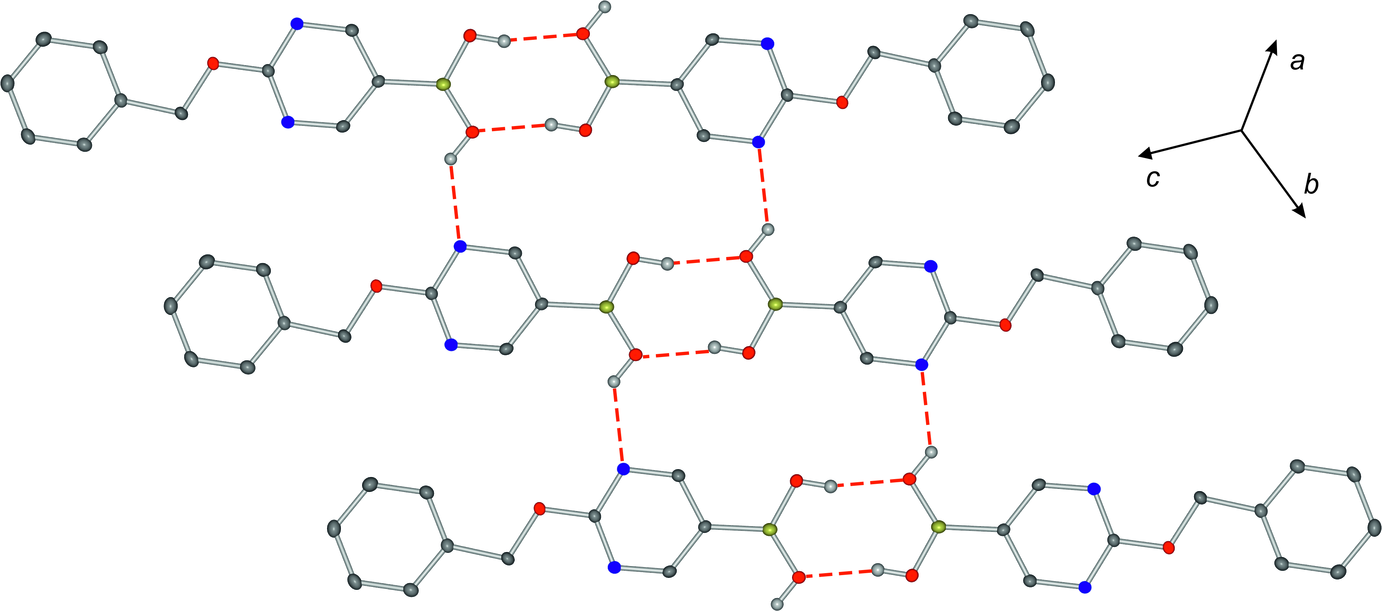

Supplement: Supplementary file 6 [file e-70-o1259-fig2.tif]

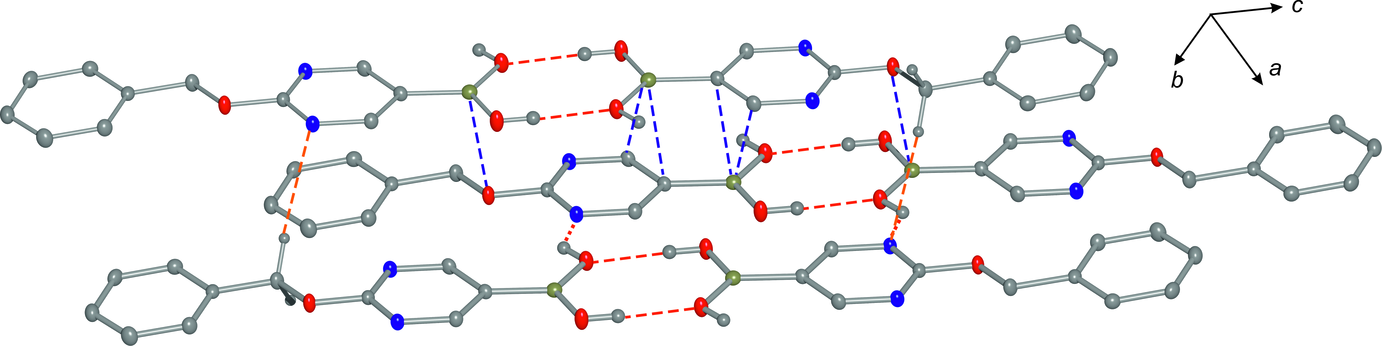

Supplement: Supplementary file 7 [file e-70-o1259-fig3.tif]
